# miR-101b Regulates Lipid Deposition and Metabolism of Primary Hepatocytes in Teleost Yellow Catfish *Pelteobagrus fulvidraco*

**DOI:** 10.3390/genes11080861

**Published:** 2020-07-29

**Authors:** Guang-Hui Chen, Tao Zhao, Xiao-Lei Wei, Dian-Guang Zhang, Mei-Qin Zhuo, Zhi Luo

**Affiliations:** 1 Key Laboratory of Freshwater Animal Breeding, Ministry of Agriculture of P.R.C., Fishery College, Huazhong Agricultural University, Wuhan 430070, China; cgh0626@webmail.hzau.edu.cn (G.-H.C.); zhaotao2017@webmail.hzau.edu.cn (T.Z.); xiaolei1205@webmail.hzau.edu.cn (X.-L.W.); ZDG@webmail.hzau.edu.cn (D.-G.Z.); zmq@mail.hzau.edu.cn (M.-Q.Z.); 2 Laboratory for Marine Fisheries Science and Food Production Processes, Qingdao National Laboratory for Marine Science and Technology, Qingdao 266237, China

**Keywords:** miR-101b, lipid deposition, metabolism, transcriptional regulation, fish

## Abstract

Excessive fat deposition in the hepatocytes, associated with excess dietary fat intake, was related to the occurrence of fatty livers in fish. miR-101b plays the important roles in controlling lipid metabolism, but the underlying mechanism at the post-transcriptional level remains unclear. The purpose of this study is to explore the roles and mechanism of miR-101b-mediating lipid deposition and metabolism in yellow catfish *Pelteobagrus fulvidraco*. We found that miR-101b directly targeted fatty acid translocase (*cd36*), caspase9 (*casp9*) and autophagy-related gene 4A (*atg4a*). Furthermore, using palmitic acid (PA) or oleic acid (OA) to incubate the primary hepatocytes of yellow catfish, we demonstrated that miR-101b inversely regulated *cd36*, *casp9,* and *atg4a* expression at the transcriptional level; the inhibition of miR-101b aggravated fatty acids (FAs, PA or OA)-induced lipid accumulation, indicating that miR-101b mediated FAs-induced variations of lipid metabolism in yellow catfish. Taken together, our study gave novel insight into the regulatory mechanism of lipid deposition and metabolism and might provide potential targets for the prevention and treatment of fatty livers in fish.

## 1. Introduction

At present, excessive hepatic lipid deposition and fatty livers are a common phenomenon for fish under the intensive aquaculture. Excessive fat intake will aggravate lipid deposition in the liver and hepatocytes, which could contribute to fatty livers and hepatic steatosis [1]. Lipid deposition, metabolism, and their regulatory processes are very complex, which involves in the balance among lipid absorption, transport, lipogenesis, and lipolysis, and several crucial enzymes and transcriptional factors participate in these metabolic processes [2]. Fatty acid translocase (CD36) is a key regulator in the FAs transport [3]. 6-phosphogluconate dehydrogenase (6PGD), glucose-6-phosphate dehydrogenase (G6PD), malic enzyme (ME) and isocitrate dehydrogenase (ICDH) play the predominant roles in the generation of NADPH, which is necessary for lipogenesis. Fatty acid synthase (FAS) is the main lipogenic enzyme that produces FAs [4]. In addition, accumulating evidences suggest apoptosis and autophagy are the pivotal pathways in regulating lipid metabolism [5,6]. Casp9 and ATG4 are the pivotal proteins involved in the apoptosis and autophagy pathways, respectively, which contributes to the control of lipid metabolism [7,8,9]. However, their regulatory mechanism remains unknown.

MicroRNAs (miRNAs) are highly conserved small non-coding RNAs with 19–25 nucleotides (nt) in length [10]. MiRNAs inhibit translation or degradation of target genes by base-pairing to the 3′-UTR of target mRNAs [11,12]. Evidences show that miRNAs are implicated in many physiological and pathological processes of fatty liver diseases [12] and act as key regulators in lipid metabolism [13,14,15]. Thus, miRNAs may be a novel therapeutic target to ameliorate fatty liver diseases.

miR-101 is an important miRNA and is considered as the biomarker for metabolic diseases, such as type 2 diabetes [16]. miR-101 has two isoforms, miR-101a and miR-101b, that are expressed differentially in many cell types. Recently, we found that miR-101b expression in the liver tissues was remarkably down-regulated in yellow catfish *Pelteobagrus fulvidraco* fed with high-fat diet (HFD) [15]. Moreover, HFD induced the dysfunction of hepatic lipid metabolism of yellow catfish. At present, the roles and mechanism of miR-101b in regulating lipid metabolism have not been investigated. The present study hypothesized that miR-101b regulated HFD-induced changes of lipid metabolism. Compared to the animal model, the cell culture of hepatocytes was considered to be a reliable model for testing metabolism for its controllability in the experimental conditions, avoidance for individual differences, and its acceptance for animal welfare [17,18]. Here, using dual-luciferase reporter assay, we determined the target genes of miR-101b in yellow catfish. Using primary hepatocytes from yellow catfish, we identified the roles and mechanism of miR-101b regulating FAs-induced variations of lipid metabolism.

## 2. Material and Methods

### 2.1. Experimental Animals and Cells

Juvenile yellow catfish were purchased from local farm (Hubei Bairong Fisheries Farm, Huanggang). HEK293 cells were obtained from the Cell Resource Center in Fishery College of Huazhong Agricultural University (HZAU). Our study followed the guideline of HZAU for the use of experimental animals and was approved by the Ethics Committee of HZAU (identification code: Fish-2016-0420, Date: 20 April 2016).

### 2.2. Validation of miR-101b Target Genes

The target genes of miR-101b were validated as described in Chen et al. [15]. At first, we synthesized wild type (WT) DNA fragments of the 3′ UTRs of *cd36*, *casp9,* and *atg4a* containing miR-101b putative binding site, and subcloned them into pmirGLO vector (Promega, USA) with the SacI and XhoI sites. Mutant (MUT) recombinant plasmids were synthesized by overlap-PCR, and the 7 mer of miR-101b Seed Sequence was mutated to ACAGTAC.

Four different treatment groups were designed: WT plasmids *+* miR-101b negative control (NC, 40 nM), WT plasmids *+* miR-101b mimics (40 nM), MUT plasmids + miR-101b NC, MUT plasmids + miR-101b mimics. The cells were incubated for 24 h, and harvested for luciferase activity assays, based on Chen et al. [15].

### 2.3. Primary Hepatocytes Culture and Treatment

The primary hepatocytes from yellow catfish were isolated and cultured following our previous studies [15,19]. Four groups were designed: Control (FA-free BCA + NC), miR-101b mimics/inhibitor (40 nM), FAs (0.25 mM PA/0.5 mM OA+NC), FAs + miR-101b mimics/inhibitor, respectively. The concentrations of FAs and miRNA inhibitor/mimics were chosen on the basis of our previous study [15]. The cells were subsequently treated for 24 h and collected for the determination of lipid deposition, enzymatic activity, and RT-qPCR analysis.

### 2.4. Sample Analysis

#### 2.4.1. Lipid Deposition

Triglyceride (TG) contents were measured using the commercial kits, based on Chen et al. [15].

Bodipy 493/503 staining was used to determine intracellular lipid droplets (LDs) on the basis of the study by Zhao et al. [20]. The intensity of fluorescence of primary hepatocytes were determined via the laser confocal microscope (Leica, Wetzlar, Germany).

#### 2.4.2. Enzymatic Activity Assays

The determination of lipogenic enzymes activities, including 6PGD, G6PD, ME, ICDH and FAS, were performed as described in Chen et al. [3]. We measured the protein content with BSA as the standard [21].

#### 2.4.3. RT-qPCR Assays

The gene expression was determined using the RT-qPCR method as reported by Chen et al. [22], and microRNAs expression was assayed by the method of stem–loop RT–PCR [16]. The specific stem-loop primers and PCR primers for each gene are shown in Table S1. To filtrate the most stable two genes as housekeeping gene using geNorm software, we detected the mRNA expression of housekeeping genes of β*-actin*, *gapdh*, *b2m*, *hprt*, *elfa*, *tbp* and *ubce.* We used the 2^−ΔΔCt^ method to calculate the relative expression of genes [23].

### 2.5. Statistical Analysis

All these data are expressed as mean ± SEM. The Shapiro-Wilk test was used to normalize all data. Bartlett’s test was used to detect the homogeneity of variances. Student’s *t*-test was used to compare the difference between two groups. *P* < 0.05 was considered to be statistically significant. SPSS19.0 software was used for the data analysis.

## 3. Results

### 3.1. miR-101b Directly Targeted cd36, casp9, and atg4a

**Figure 1:**
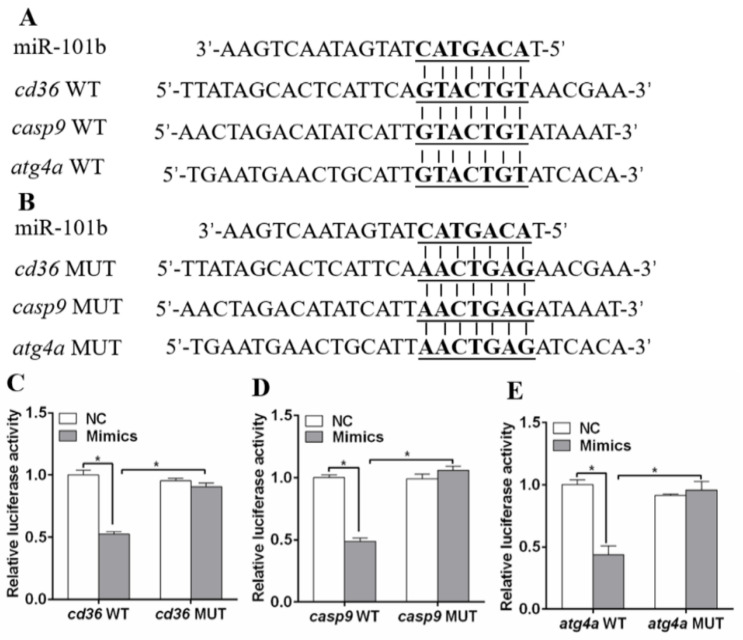
miR-101b targeted *cd36*, *casp9* and *atg4a* in yellow catfish. (**A**,**B**) Schematic representation of wild type (WT) and mutant (MUT) for the miR-101b target sequence within the 3′UTR of *cd36*, *casp9,* and *atg4a* of yellow catfish. Seed sequences are highlighted in bold. (**C**–**E**) The luciferase activities of the reporter of *cd36*, *casp9,* and *atg4a* of yellow catfish. Results are expressed as the mean ± SEM, *n* = 3. * *P* < 0.05 (Student’s *t* test).

We used Targetscan Fish 6.2 and miRWalk 3.0 to identify target genes of miR-101b. Figure 1A showed that the 3′UTRs of *cd36*, *casp9* and *atg4a* of yellow catfish possessed the potential miR-101b binding sites. Compared to the NC, miR-101b mimics dramatically suppressed the luciferase activities of pmirGLO-WT-*cd36,* pmirGLO-WT-*casp9* and pmirGLO-WT-*atg4a* in HEK-293T cells, while the luciferase activity of pmirGLO-MUT-*cd36,* pmirGLO-MUT-*casp9* and pmirGLO-MUT-*atg4a* were rescued (Figure 1C–E). These results suggested that miR-101b could directly target *cd36*, *casp9* and *atg4a*.

### 3.2. miR-101b Inversely Regulated cd36, casp9, and atg4a Expression in Hepatocytes of Yellow Catfish

**Figure 2:**
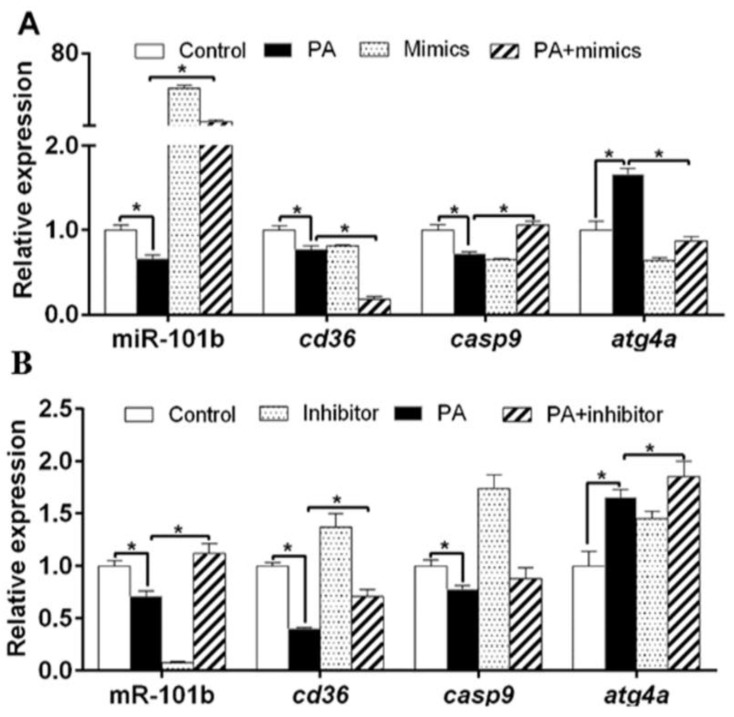
The influences of PA and miR-101b mimics (**A**) or inhibitor (**B**) co-incubation on gene expression of miR-101b and its target genes in yellow catfish. Results are expressed as the mean ± SEM, *n* = 3. * *P* < 0.05 (Student’s *t* test).

PA down-regulated miR-101b, *cd36* and *casp9* expression, but up-regulated the gene concentrations of a*tg4a* (Figure 2A). Compared to PA group, miR-101b mimics and PA co-incubation up-regulated gene concentrations of miR-101b and *casp9*, but down-regulated of gene concentrations of *cd36* and *atg4a*. Compared to PA group, co-treatment with miR-101b inhibitor and PA up-regulated gene concentrations of miR-101b, *cd36* and *atg4a* (Figure 2B).

**Figure 3:**
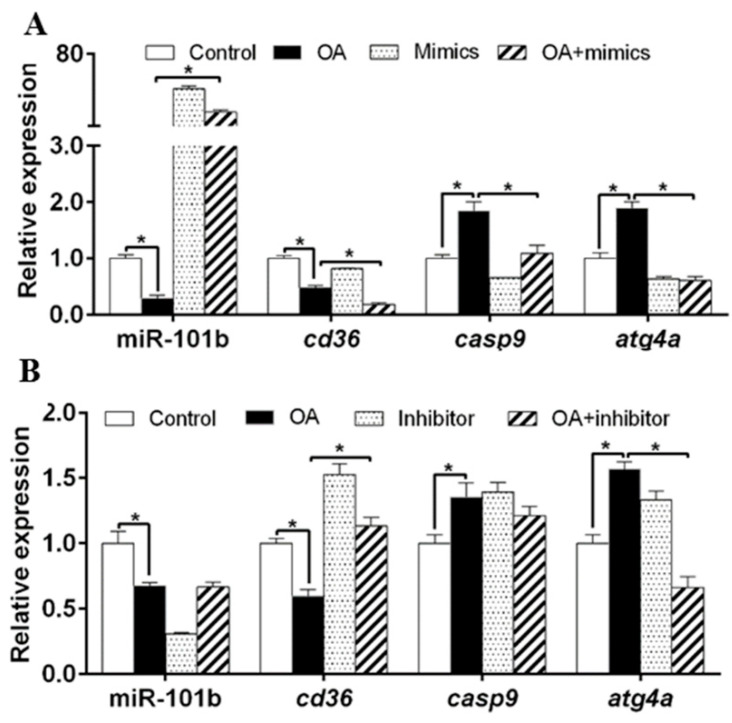
The influences of oleic acid (OA) and miR-101b mimics (**A**) or inhibitor (**B**) co-incubation on gene expression of miR-101b and its target genes in yellow catfish. Results are expressed as the mean ± SEM, *n* = 3. * *P* < 0.05 (Student’s *t* test).

OA incubation down-regulated miR-101b and *cd36* expression, but up-regulated gene concentrations of *atg4a* and *casp9* (Figure 3A). Compared to OA group, co-treatment with miR-101b mimics and OA up-regulated miR-101b concentration, but down-regulated gene concentrations of *cd36*, *casp9* and *atg4a*. Compared to OA group, co-treatment with miR-101b inhibitor and OA up-regulated expression level of *cd36*, but down-regulated expression level of *atg4a* (Figure 3B). Altogether, these results indicated that miR-101b mediated the FA-induced changes of *cd36*, *casp9* and *atg4a* expression.

### 3.3. miR-101b Mediated PA-Induced Changes of Lipid Metabolism in Hepatocytes of Yellow Catfish

**Figure 4:**
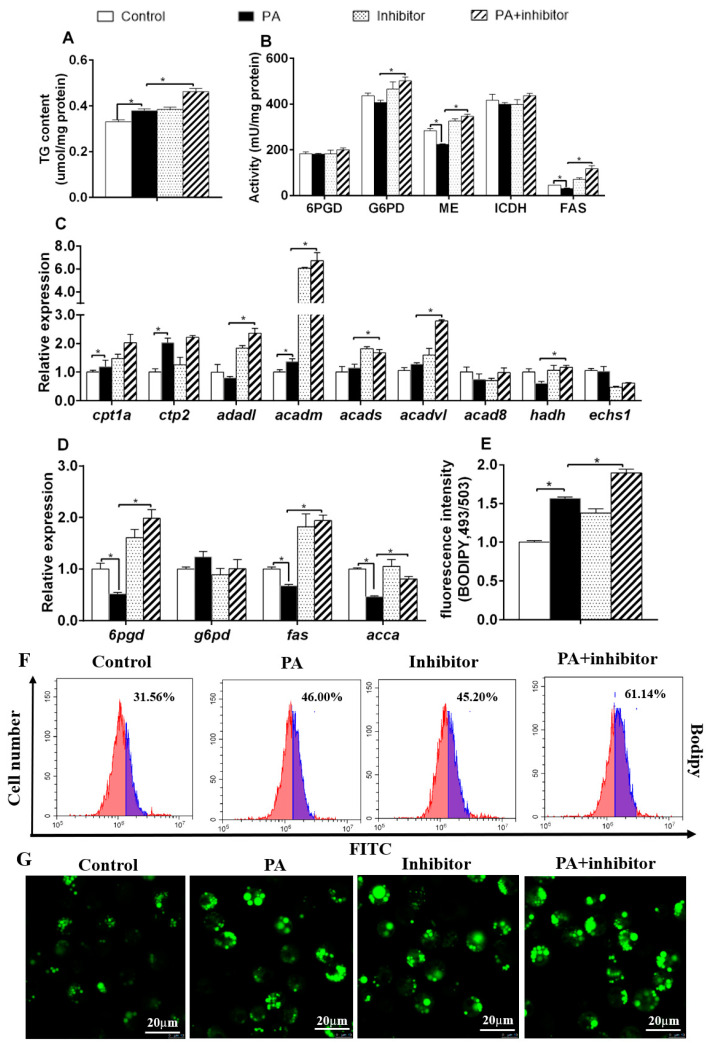
The influences of PA and miR-101b inhibitor co-incubation on lipid deposition in hepatocytes of yellow catfish. (**A**) TG content; (**B**) lipogenic activities; (**C**,**D**) mRNA expression of lipid metabolism; (**E**) fluorescence intensity after Bodipy 493/503 staining; (**F**) flow cytometric analysis of Bodipy 493/503 staining; (**G**) the images of Bodipy 493/503–stained LDs; Scale bars, 20 μm. Results are expressed as the mean ± SEM, *n* = 3. * *P* < 0.05 (Student’s *t* test).

PA incubation increased TG content, and up-regulated *cpt1a*, *cpt2,* and *acadm*, mRNA expression, but reduced ME and FAS activities, and down-regulated mRNA contents of *6pgd*, *fas* and *acca* (Figure 4A–D). Compared to PA group, co-treatment with miR-101b inhibitor and PA increased TG content, and G6PD, ME and FAS activities, and mRNA contents of *6pgd*, *fas*, *acca* and *cd36*, together with mRNA contents of *acadl*, *acadm*, *acads*, *acadvl* and *hadh*. miR-101b inhibitor pretreatment aggravated PA-induced increase of fluorescence intensity, as shown by BODIPY 493/503 staining (Figure 4E–G). Thus, miR-101b mediated PA-induced variations of lipid metabolism in hepatocytes of yellow catfish.

### 3.4. miR-101b Mediated OA-Induced Changes of Lipid Metabolism in Primary Hepatocytes of Yellow Catfish

**Figure 5:**
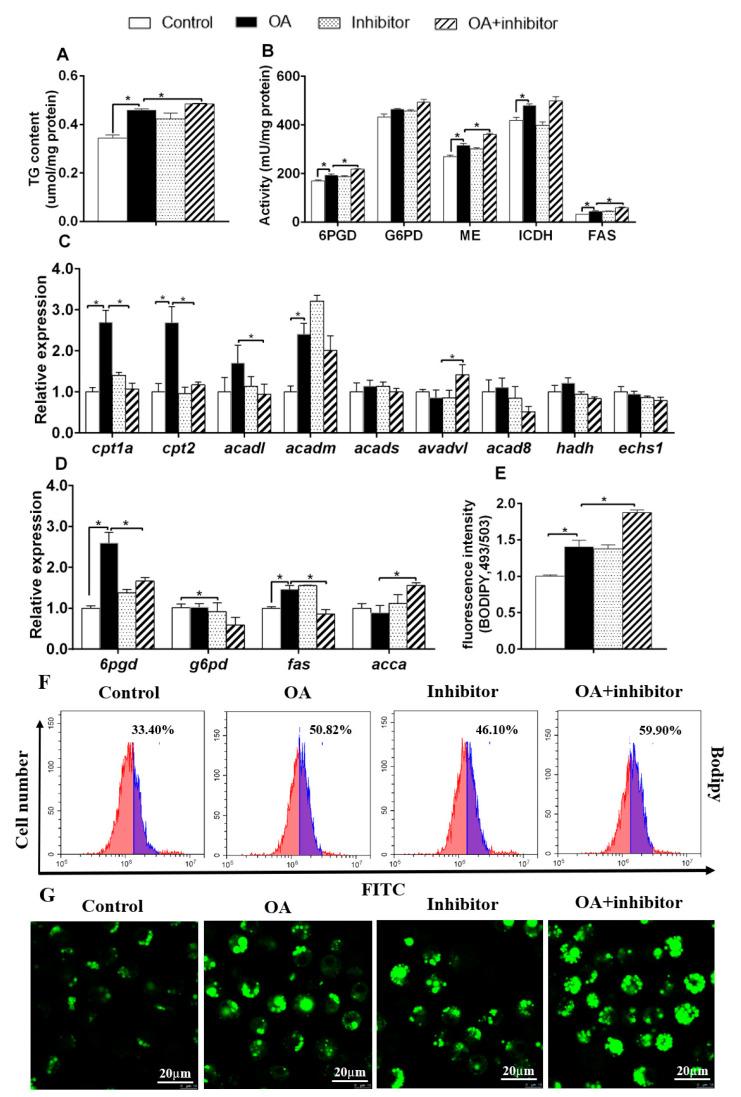
The influences of OA and miR-101b inhibitor co-incubation on lipid deposition in the hepatocytes of yellow catfish. (**A**) TG content; (**B**) lipogenic activities; (**C**,**D**) mRNA expression of lipid metabolism; (**E**) fluorescence intensity after Bodipy 493/503 staining; (**F**) flow cytometric analysis of Bodipy 493/503 staining; (**G**) the images of Bodipy 493/503–stained LDs; Scale bars, 20 μm. Results are expressed as the mean ± SEM, *n* = 3. * *P* < 0.05 (Student’s *t* test).

OA incubation increased TG content, up-regulated activities of 6PGD, ME, ICDH and FAS, and mRNA contents of *6pgd*, *fas*, *cpt1a*, *cpt2* and *acadm* (Figure 5A–D). Compared to OA group, miR-101b inhibitor and OA co-treatment decreased increased TG content, G6PD, ME and FAS activities, and *acca* and *adadl* mRNA expression*,* but decreased mRNA expression of *6pgd*, *g6pd* and *fas,* together with mRNA expression of *cpt1a*, *cpt2* and *acadl*. miR-101b inhibitor pretreatment also aggravated OA-induced increase of fluorescence intensity, as shown by BDIPY 493/503 staining (Figure 5E–G). Thus, miR-101b mediated OA-induced variations of lipid metabolism in hepatocytes of yellow catfish.

## 4. Discussion

Accumulating evidences have shown that miRNAs are implicated in the lipid metabolism [14,15,24], suggesting that miRNAs may be a novel therapeutic target to fatty livers. Thus, it is rather crucial to identify novel miRNAs that target key nodes of lipid metabolism and explore their regulatory mechanisms.

In the present study, we found that miR-101b directly targeted *cd36*, *casp9* and *atg4a* by using reporter gene analysis. Moreover, miR-101b inhibitor elevated gene concentrations of *cd36*, *casp9,* and *atg4a* in hepatocytes of yellow catfish, suggesting that miR-101b inversely controlled the expression of *cd36*, *casp9* and *atg4a*. CD36, also called fatty acid translocase, is important in the FAs transporters [25]. Increased expression of *cd36* was beneficial for absorption of FAs. Four ATG4 subtypes have been identified in mammals, and ATG4a protein could regulate the deconjugation of LC3, which ultimately affects the amplitude of the autophagic response [26,27]. Elevated expression of *atg4a* could promote the formation of autophagosomes, which potentially influenced lipid metabolism [7,20]. Casp9 plays a central role in the mitochondrial apoptotic pathway [28], which regulates lipolysis [8]. Interestingly, PA + mimics/OA + mimics treatment in hepatocytes led to a robust reduction in expression of cd36 but increased the expression of *cas9* and *atg4a*. We suspected that these genes were involved in the different roles of lipid metabolism, and it required further exploration. Similarly, studies found that ATG4D was the target gene of miR-101 [29,30].

To further investigate the mechanism of miR-101b-mediating FAs-induced lipid deposition in hepatocytes, we determined the effects of FAs and miR-101b inhibitor on lipid deposition and metabolism. In the diet, serum and liver tissue, PA (a saturated fatty acid) and OA (a monounsaturated fatty) are two of the most abundant FAs [31]. We conducted the experiment by the co-treatment with miR-101b inhibitor/mimics and OA/PA in hepatocytes of yellow catfish, respectively. In the present study, miR-101b mimics pretreatment aggravated OA/PA-induced down-regulation of *cd36* mRNA concentration but miR-101b inhibitor pretreatment alleviated OA/PA-induced down-regulation of *cd36* mRNA concentration. Given that CD36 played the crucial roles in lipid metabolism [25], our data established miR-101b was a critical regulator of hepatic lipid metabolism by directly targeting *cd36*. On the other hand, we observed that higher hepatic TG level, associated with elevated lipogenic activities and gene expression in miR-101b interfering hepatocytes of yellow catfish, strongly indicating that miR-101b was required for normal lipid homeostasis. Unexpectedly, PA/OA reduces the expression of CD36 in hepatocytes. We suspected that there may be a negative feedback mechanism that the excessive accumulation of TG in hepatocytes inhibited the expression of CD36, which ultimately inhibited the absorption of fatty acids, as reported in other studies [32].

In the present study, PA increased TG content, but reduced ME and FAS activities, and mRNA concentrations of *6pgd*, *fas*, *acca* and *cd36*. Studies in our laboratory pointed out that PA elevated NEFA content and regulated gene expression of lipid absorption in hepatocytes of yellow catfish [33]. The decreased gene expression and enzymatic activities related to lipid absorption as well as lipogenesis contributed to suppress lipid accumulation [3]. The PA-induced suppression in gene expression and activities of lipid absorption and lipogenic enzymes may be associated with the negative feedback regulation caused by excessive lipid deposition in hepatocytes and the protection against the liptoxicity, as suggested by several authors [34,35]. The PA-induced increase of TG content in miR-101b inhibitor + PA group was aggravated, suggesting that miR-101b inhibitor could accelerate the synthesis process from FAs to TG. In addition, miR-101b inhibitor reversed the reduction of G6PD, ME and FAS activities, and mRNA expression of *ca36*, *6pgd*, *fas* and *acca* induced by PA. Lipid absorption and lipogenesis are the fundamental regulatory way in lipid metabolism [3]. Thus, our results demonstrated that the miR-101b regulated PA-induced lipid metabolism via lipid absorption and lipogenesis. On the other hand, the present study showed that OA incubation increased TG content, up-regulated 6PGD, ME, ICDH and FAS activities, and mRNA concentrations of *6pgd* and *fas*, but down-regulated *cd36* mRNA concentration. Previous studies pointed out that OA improved FA and TG synthesis via lipogenesis, thereby inducing lipid accumulation [36,37]. We found that miR-101b inhibitor aggravated OA-induced increase of TG content, G6PD, ME, and FAS activities, and mRNA concentrations of *6pgd*, *g6pd* and *fas,* suggesting that miR-101b mediated OA-induced changes of lipid metabolism indirectly via these enzymes and genes. In addition, flow cytometry and confocal microscopy analysis indicated that miR-101b inhibitor pretreatment aggravated the PA or OA-induced increase in the volume and amounts of LDs in hepatocytes. Here, we provided in vitro data to demonstrate that miR-101b was a key mediator of lipid metabolism in yellow catfish.

In the present study, PA and OA could promote the expression level of genes involved in fatty acid β-oxidation. Wu et al. [9] found that HFD and FAs incubation elevated the capacity of fatty acid β-oxidation. Here, although PA and OA increased TG content, PA and OA produced the adverse effect on lipogenic activities and gene expression. Similarly, other studied found that PA and OA produced different effects on lipid metabolism in several hepatocytes [38,39]. Therefore, different sources of FAs induced the different capacity for lipid synthesis, as reported by other workers [38,40]. Moreover, Mei et al. [38] pointed out that OA, as the unsaturated fatty acid, could promote the formation of TG-enriched LDs, but PA, as the saturated fatty acid, had the poor conversion into LDs. Zámbó et al. [41] found that the deposition of FAs was deemed to possess higher lipotoxicity and more harmful to hepatocytes than TG. Thus, the hepatocytes could readily incorporate FAs into cytoplasmic TGs, which might protect cells against their lipotoxicity [32,40].

In conclusion, miR-101b directly targeted *cd36*, *casp9,* and *atg4a*; miR-101b inversely regulated *cd36*, *casp9,* and *atg4a* expression at the transcriptional level; miR-101b mediated FAs-induced variations of lipid metabolism through directly targeting *cd36*; PA and OA incubation induced different effects on lipid metabolism of hepatocytes. Taken together, we determined the roles of miR-101b mediating FAs-induced changes of lipid metabolism, which might help further our understanding of mechanisms that contribute to the prevention and treatment of fatty liver and its related metabolic disorders.

## Supplementary Materials

The following are available online at https://www.mdpi.com/2073-4425/11/8/861/s1, Table S1: Primers used for qRT-PCR analysis.
