# Supplementary material for: miR-101b Regulates Lipid Deposition and Metabolism of Primary Hepatocytes in Teleost Yellow Catfish Pelteobagrus fulvidraco
Source: Genes (Basel). 2020 Jul 29;11(8):861. doi: 10.3390/genes11080861 (PMC7463558; doi:10.3390/genes11080861)
Supplement: Supplementary file 1 [file genes-11-00861-s001.pdf]

genes

Table S1. Primers used for qRT-PCR analysis.

Genes	Forward primer (5'-3')	Reverse primer (5'-3')	Accessin No.
miR-101b	TCGGCAGGTACAGTACTATGAT	CTCAACTGGTGTCGTGGAGTC	
6pgd	GCTCTGATGTGGCGAGGTGG	CGTAGAAGGACAGTGCAGTGG	JX992745
g6pd	CAGGAATGAACGCTGGGATG	TCTGCTACGGTAGGTCAGGTCC	JX992744
fas	AACTAAAGGCTGCTGGTTGCTA	CACCTTCCCGTCACAAACCTC	JN579124
acca	GGGGTTTTCACGCTGCTTC	GGTTCTGATTGGGTCGTCCTG	JX992746
cd36	GATCGTTCTGATTTCGGTTGG	TTATTGTCGTACTTCGGCACTG	MG574317
atg4a	ATGGAGGCAGTTTTAGCCAAGTAT	TGTATGTAAACCACAGCCGTGAA	KY062775
casp9	CACAGCACCAAGGCTAGATGA	TCCTGGAAATGTTGAGTAGGACA	KY072821.1
cpt1a	ATTTGAAGAAGCACCCAGAGTATGT	CCCTTTTATGGACGGAGACAGA	JQ074177
cpt2	AAAGTTTACCGAGATTGCCAGTTCC	TCACGAGCCAGTTTCTCCGTTTTA	MG599811
acadl	TAACGGCTGGATGAGTGACCTGG	GCTATTATCAACCGCTCCTGTGGC	MG599805
acadm	GCAGAAGGAGTTCCAGGAGGTGTC	CAGCAATAATGACCGGCATTTGTC	MG599804
acads	AAGTAGGCTGCTTTGCTTTGAGTGAG	AATTGGTGATCCAGGCTTTGGTG	MG599806
acadvl	CGAGACCTGCGAATCTTCCGTAT	AATCTCCCCAGCCAACAAACCAG	MG599809
acad8	ACCATTACATCCTGAACGGCTCCA	GCGAGTTCCAACCAACCTTCTTCT	MG599807
hadh	GCTTGACGACAGGTTTCGGGTTT	TGTTTAGCTGCTTATCCTTTGATTTGG	MG599815
echs1	ACAGGATGCCAAACAGTCTGGTCTT	TCTTCTCCAACCGATTGCCTTCA	MG599820
β-actin	GCACAGTAAAGGCGTTGTGA	ACATCTGCTGGAAGGTGGAC	EU161066
tbp	AGCAAAGAGTGAGGAGCAGT	ACTGCTGATGGGTGAGAACA	KP938525
elfa	GTCTGGAGATGCTGCCATTG	AGCCTTCTTCTCAACGCTCT	KU886307
b2m	GCTGATCTGCCATGTGAGTG	TGTCTGACACTGCAGCTGTA	KP938520
hprt	ATGCTTCTGACCTGGAACGT	TTGCGGTTCAGTGCTTTGAT	KP938523
ubce	TCAAGAAGAGCCAGTGGAGG	TAGGGGTAGTCGATGGGGAA	KP938524

gapdh	TTTCAGCGAGAGAGACCCAG	ATGACTCTCTTGGCACCTCC	KP938521
u6	CTCGCTTCGGCAGCACA	AACGCTTCACGAATTTGCGT	NM_00103460
